# Light-dark rhythms during incubation of broiler chicken embryos and their effects on embryonic and post hatch leg bone development

**DOI:** 10.1371/journal.pone.0210886

**Published:** 2019-01-25

**Authors:** Carla W. van der Pol, Inge A. M. van Roovert-Reijrink, Conny M. Maatjens, Sander W. S. Gussekloo, Sander Kranenbarg, Jan Wijnen, Remco P. M. Pieters, Henk Schipper, Bas Kemp, Henry van den Brand

**Affiliations:** 1 Research department, HatchTech B.V., Veenendaal, the Netherlands; 2 Adaptation Physiology Group, Wageningen University and Research, Wageningen, the Netherlands; 3 Experimental Zoology Group, Wageningen University and Research, Wageningen, the Netherlands; University of Sassari, ITALY

## Abstract

There are indications that lighting schedules applied during incubation can affect leg health at hatching and during rearing. The current experiment studied effects of lighting schedule: continuous light (24L), 12 hours of light, followed by 12 hours of darkness (12L:12D), or continuous darkness (24D) throughout incubation of broiler chicken eggs on the development and strength of leg bones, and the role of selected hormones in bone development. In the tibiatarsus and femur, growth and ossification during incubation and size and microstructure at day (D)0, D21, and D35 post hatching were measured. Plasma melatonin, growth hormone, and IGF-I were determined perinatally. Incidence of tibial dyschondroplasia, a leg pathology resulting from poor ossification at the bone’s epiphyseal plates, was determined at slaughter on D35. 24L resulted in lower embryonic ossification at embryonic day (E)13 and E14, and lower femur length, and lower tibiatarsus weight, length, cortical area, second moment of area around the minor axis, and mean cortical thickness at hatching on D0 compared to 12L:12D especially. Results were long term, with lower femur weight and tibiatarsus length, cortical and medullary area of the tibiatarsus, and second moment of area around the minor axis, and a higher incidence of tibial dyschondroplasia for 24L. Growth hormone at D0 was higher for 24D than for 12L:12D, with 24L intermediate, but plasma melatonin and IGF-I did not differ between treatments, and the role of plasma melatonin, IGF-I, and growth hormone in this process was therefore not clear. To conclude, in the current experiment, 24L during incubation of chicken eggs had a detrimental effect on embryonic leg bone development and later life leg bone strength compared to 24D and 12L:12D, while the light-dark rhythm of 12L:12D may have a stimulating effect on leg health.

## Introduction

In nature, chicken eggs will be exposed to short bouts of light when the hen leaves the nest to eat and drink [1]. In commercial practice, eggs are incubated in complete darkness, except for the moment the eggs are candled and transferred from the setter machine, where they are incubated on egg trays for the first 18 days, to the hatcher, where eggs are allowed to hatch in baskets for the last 3 days of incubation. However, there is evidence to suggest that by providing light during the incubation process, a positive effect on embryonic development may occur [2–5].

One effect that has been observed in other studies is that lighted incubation may stimulate leg bone development and leg health in meat type chickens (“broilers”). [6] found 2.2% fewer chicks that were too weak to stand or suffered from leg abnormalities at the moment of hatching in embryos incubated under 12h of light, followed by 12h of darkness (12L:12D) compared to continuous darkness (24D) Furthermore, they found higher developmental instability, based on composite asymmetry of the leg bones, at day 14 post hatching for broilers incubated under 24D than under 12L:12D [6].

Embryonic bone development involves several processes which may alter leg health and bone development at hatch and in later life. Chicken embryo bones are first formed as a cartilage model [7], which later becomes ossified through a ring of bone material at the mid-diaphysis of the bone [8,9]. At the epiphyseal ends of the long bones, an epiphyseal or growth plate can be observed, where bone length is realized through production and ossification of cartilage. Cartilage cells, called chondrocytes, can be observed in resting, proliferating, and hypertrophic zones, after which they become apoptotic and their matrix becomes calcified by bone cells called osteoblasts to form bone material [10,11]. In the current experiment, bone development is studied through embryonic ossification, embryonic and post hatch bone dimensions, and post hatch bone microstructure. These are used as a reflection of developmental processes in the bone. Additionally, bone dimensions and microstructure may provide insight into bone strength [12].

It is not clearly known which physiological pathways are involved in the relationship between lighting schedule and bone development. It was speculated that exposing embryos to a light-dark schedule would create a circadian rhythm in levels of melatonin [2], a hormone known to stimulate bone development in mammals [13]. A relationship between melatonin and bone development in chickens has also been suggested [14]. Chickens that were pinealectomised at 3 days of age (eliminating circulating melatonin) all developed scoliosis within two weeks. Scoliosis incidence in pinealectomised chickens was reduced to 20% when melatonin injections were administered every other day [14]. [15] found disrupted endochondral ossification at the epiphyseal plate for pinealectomised chickens. Melatonin might have both a direct effect on bone development and an indirect effect by stimulation or inhibition of other hormones involved in bone development. In chickens, melatonin stimulates hypothalamic GH release [16], and GH and IGF-I were observed to peak along with darkness-dependent melatonin release [17]. GH and IGF-I both have a stimulatory effect on proliferation of cartilage cells (called chondrocytes) in the growth plates of long bones [18,19].

Broilers are especially interesting in the study of bone development because the incidence of leg bone pathologies in the rearing period is higher than in other chicken populations as a result of their high growth rate [20,21]. Developmental leg bone pathologies, such as tibial dyschondroplasia (in which cartilage fails to mature and is not replaced by bone), can be prevalent in up to 57.1% of a flock [22]. We hypothesize that developmental leg abnormalities can partly be prevented by providing a growing broiler embryo with optimal circumstances for leg bone development. For example, incidence of tibial dyschondroplasia has been proven to be increased when incubation temperatures are above or below 37.8°C in the first week of incubation [23]. Since a high incidence of tibial dyschondroplasia may be considered to be an indicator of suboptimal embryonic leg bone development, it is used as a readout parameter in the current experiment.

Since [1] found improved leg health at hatching and 14 days after hatching for broilers exposed to 12L:12D compared to 24D, we expect that the stimulatory effects of 12L:12D during incubation compared to 24L can already be seen in embryonic bone development. Bone development is reflected here by heavier, longer, or thicker leg bones, and earlier onset or an increased rate of developmental processes such as ossification. If, indeed, melatonin is involved as a pathway to alter bone development, we expect that 12L:12D will result in better bone development and leg health than continuous light (24L), since melatonin release is darkness dependent. We expect that these effects are long term and can be observed till slaughter age in better developed, healthier bones.

The current experiment aims to investigate effects of light-dark rhythms (24D, 24L, or 12L:12D) applied throughout incubation of broiler chicken embryos on embryonic plasma melatonin levels, GH and IGF-I at hatch, leg bone development during incubation, at hatch, and at slaughter age (5 week post hatch), and incidence of tibial dyschondroplasia at slaughter age. The role of embryonic plasma melatonin levels, GH and IGF-I at hatch are investigated as a potential pathway for altered bone development.

## Materials and methods

The experimental design and protocol were approved by the Institutional Animal Care and Use Committee of Wageningen University, Wageningen, the Netherlands.

### Incubation

#### Experimental setup

Freshly laid Ross 308 broiler chicken eggs from a 40 week old parent flock were selected for egg weights between 62 and 65 grams. 744 embryos and chickens were used for the current experiment. Eggs were stored at 18°C for 3 days on egg trays in a commercial hatchery (Lagerwey BV, Lunteren, the Netherlands) before transportation to the experimental facilities of Wageningen University & Research (Wageningen, the Netherlands). Upon arrival, eggs were placed in one climate respiration chamber per treatment. In climate respiration chambers, climate conditions such as temperature, relative humidity, and CO_2_ levels can be controlled strictly in an airtight situation [24]). Three regimes with 310 eggs each (to allow for an estimated hatchability of 80%) were used for incubation: 24L; an intermittent light-dark schedule (12L:12D) that started with the light period on embryonic day (E)0; or 24D. Lighting schedules were applied from E0 until hatch. Light was provided by 3 white light emitting diode (LED) strips per egg tray of approximately 500 lux at egg level and a colour temperature of 6,050K (wavelength range 420 to 780 nm, peak at 454 nm). Until E19 + 6h, eggshell temperature was measured using temperature sensors (NTC Thermistors: type DC 95; Thermometrics, Somerset, UK) attached to 5 randomly chosen eggs per treatment. Sensors were attached to the equator of the egg using a small piece of duct tape and heat conducting paste (Dow Corning 340 Heat Sink Compound, Dow Corning GmbH, Wiesbaden, Germany) facing away from the light source. Air temperature was measured continuously using 5 1/3 DIN-B PT100 temperature sensors with an accuracy of ≤± 0.1°C per incubation chamber. Air temperature was adjusted automatically to maintain a constant eggshell temperature of 37.8°C throughout incubation to ensure that there was no temperature effect of the lighting treatments at embryo level. From E19 +6h onward, the climate respiration chamber’s temperature was fixed at the current air temperature, and eggshell temperatures were allowed to increase during the hatching process. Relative humidity was measured using Novasina Hygrodat 100 with HIA-13/20M combi sensors and maintained between 45 and 55% throughout incubation. CO_2_ concentration was measured continuously using two gas flow meters (Itron Delta 2050 MP series, type G100) per incubation chamber with an accuracy within ±0.5%. CO_2_ concentration did not exceed 0.35%. Until E18, eggs were incubated on egg trays. At the start of incubation, trays were turned to an angle of 45° from the horizontal position, and trays were then turned hourly to an angle of 45° until E18. From E18 onward, eggs were placed in baskets for hatching, and no longer turned.

#### Measurements

**Fig 1:**
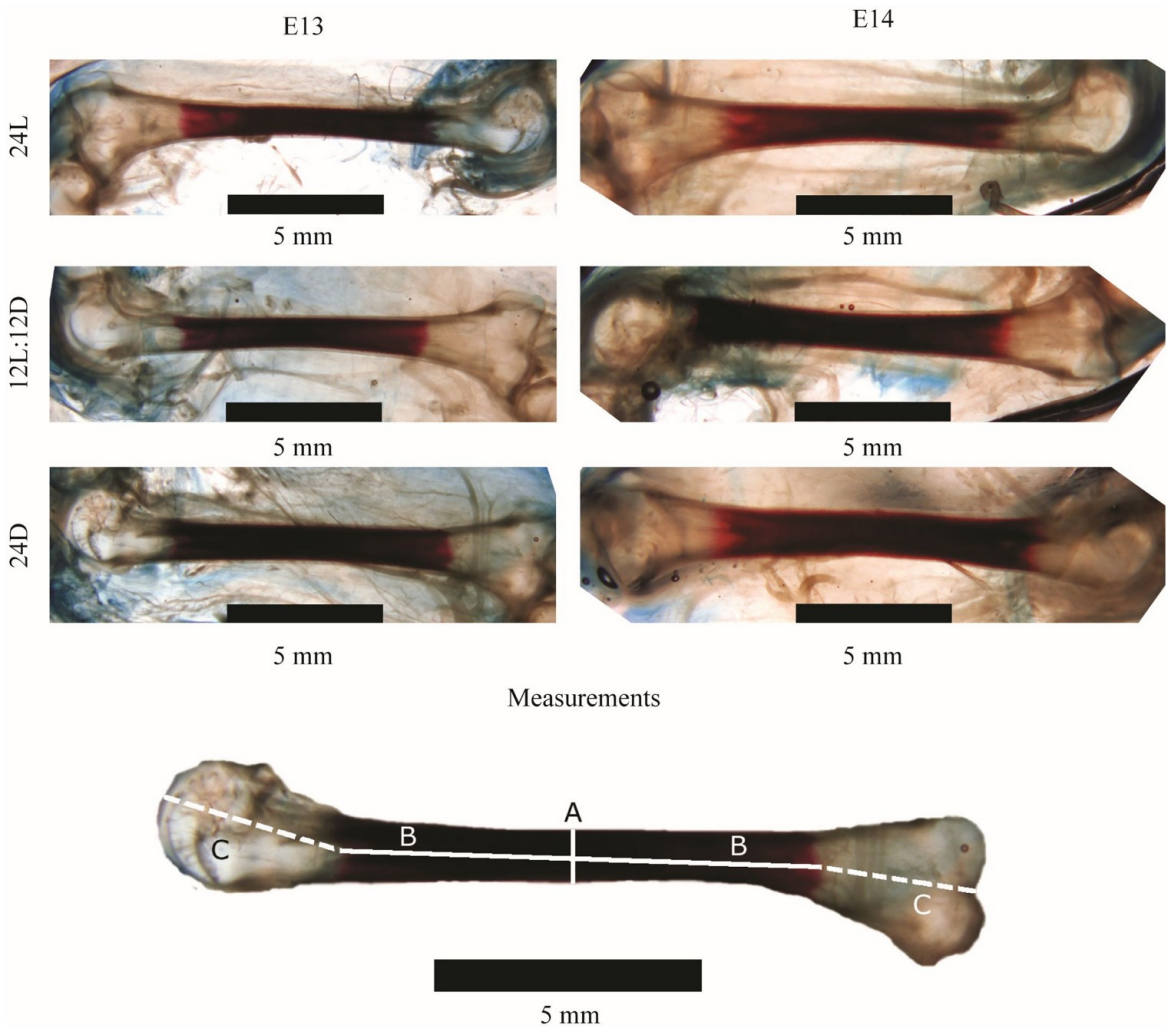
Examples of tibiatarsi stained with Alizarin red and Alcian blue staining in a procedure modified from [24] on embryonic day (E)13 and E14 in broiler embryos incubated under 24L, 12L:12D, or 24D of white LED light from day 0 until hatching. A = bone width; B = ossified length; B + C = total length. The ossified part of the bone is stained red; the cartilaginous part of the bone is uncoloured. The black bar indicates the scale (5 mm).

From E8 until E14, 10 eggs per day, per treatment (N = 210) were removed from the incubator for measurement of ossification of the femur and tibiatarsus, using Alizarin red and Alcian blue staining in a procedure modified from [25]. The time frame of E8 till E14 was chosen because [7] demonstrated that ossification in the tibiatarsus and femur starts by E9, and we expected the largest portion of ossification to be completed by E14. Eggs were first sampled from the 12L:12D treatment, then 24L, then 24D. Total sampling time was approximately 20 min per treatment. Embryos were fixed in formalin for 24 h, and then stored for 10 months in 70% alcohol solution for later analysis. For the E13 and E14 embryos, most of the leg muscle and skin were removed before storing to facilitate penetration of the staining solutions. Embryos were placed in 96% alcohol for 24 h prior to the staining process. They were then transferred to a Alcian blue solution (80 mL 100% alcohol, 20 mL acetic acid, and 10 mg Alcian blue 8GS) for 24 h for embryos aged E8 to E10 or 48 h for embryos aged E11 to E14. Alcian blue staining was performed to provide a clear contrast between the cartilage and surrounding soft tissue. This cartilage staining did not take very well, but the difference between cartilage and surrounding tissue was still visible. Embryos were then placed in alcohol solutions that decreased every 2 h from 96% alcohol at the start to 100% MiliQ water after 10 h. Embryos were transferred to a Trypsin solution (30 mL saturated sodium borate water, 70 mL MiliQ water, and 1 g Trypsin) 2h. Embryos were then placed in an Alizarin red solution (100 mL 0.5% KOH and 10 mg Alizarin red S. (A16)) for 24 h, and then in the Trypsin solution until the stained bones were visible, which ranged from 11 days for E8 embryos to 20 days for E14 embryos. Embryos were then placed in a 0.5% KOH-Glycerine solution with 3–4 drops of 3% H_2_O_2_/100 mL solution in a KOH: Glycerine ratio of 3: 1 for 1 day, 1: 1 for 2 days, and 1: 3 for 3 days. Finally, all embryos were placed in 100% glycerine with a few thymol crystals. Embryos were placed in a petri dish and photographed through a Zeiss StemiSV 11 stereomicroscope (Jena, Germany) with an attached Olympus DP50 camera (Tokyo, Japan), and Olympus AnalySIS-FIVE software (Tokyo, Japan). Leg bones were photographed with a 0.63x objective, at a 6.6 x 10 magnification, with a 2.5x adapter. For examples of images of the stained bones, see Fig 1. The total length, ossified length, and width (at the middle of the diaphysis) of the femur and tibiatarsus of each left and right leg of the embryos were measured. Because bones were often not completely straight, the line for total length was drawn in three parts for standardisation; from the top of the bone’s proximal end to the ossified zone, through the ossified zone, to the dent in the bone’s distal end (Fig 1). Ossified percentage was calculated as ossified length / total length x 100.

On E18 +12h (444 h of incubation), E18 +18h (450 h of incubation), E19 (456 h of incubation), and E19 +6h (462 h of incubation), plasma melatonin was measured in 5 embryos per treatment (N = 60). Sampling took approximately 30 minutes per time point. The first measurement took place immediately prior to the start of 12L:12D’s dark period, the second measurement in the middle of the dark period, the third measurement immediately prior to the start of the light period, and the fourth measurement in the middle of the light period. Embryos were removed from the eggshell first, and blood was collected from their jugular vein using a 1 mL syringe and 30-gauge needle into heparin coated tubes before decapitation. Blood was centrifuged for 10 min at 12,000 rpm. After centrifugation, plasma was collected and stored at -20°C. Melatonin was later analysed by a double antibody sandwich technique (Chicken melatonin ELISA, catalogue no. MBS262913, MybioSource, CA, USA).

At E19 +12h, the hatching process started, and hatchlings were checked every 3 h. Chickens that were assigned to be sampled immediately (every 3^rd^ chick that hatched; 63 per treatment; N = 180) were removed from the climate respiration chambers within 3 h post hatch. They were decapitated, and their blood was collected for IGF-I and GH analysis in 63 chickens that hatched in the middle of the hatch window (at 492, 495, and 498 h of incubation, which coincided with 12L:12D’s light period). Blood was centrifuged for 10 min, at 12,000 rpm. After centrifugation, plasma was collected and stored at -20°C. IGF-I was later analysed using a chicken enzyme-linked immunosorbent assay kit (SEA050Ga 96; Cloud-Clone Corp., Texas, USA), and growth hormone was later analysed using a chicken ELISA kit (CSB-E09866Ch; Cusabio, Maryland, USA). Non-bone tissue of the legs of the decapitated chickens was removed, and the femur and tibiatarsus were weighed. Bones were then wrapped in gauze soaked in saline, and stored in a freezer at -20°C for later analysis.

### Post hatch

#### Experimental setup

After 21 days and 12h of incubation, all hatched chickens that had been assigned to post hatching measurements (108 per treatment; N = 324) were transported to Coppens’ Poultry Research Centre (Vlierden, the Netherlands) for 1h in a climate controlled car. They were housed in floor pens measuring 0.95 x 1.55 m with 9 pens per incubation treatment. From placement until D21 post hatch, 12 chickens were housed per pen. On D21, half of the chickens were removed for sampling, and 6 chickens per pen remained there until final sampling at D35. On D0, all chickens were exposed to 24 h of light before starting a lighting schedule of 16L:8D. The air temperature was maintained at 33°C at D0, decreasing linearly to 19°C at D35. Wood shavings were used as bedding of the pens, and water from nipple drinkers and a commercially available feed were provided *ad libitum*.

#### Measurements

At hatch, chicks designated for further growth (108 per treatment; N = 324) received an individual number on a neck label. At E21 +12h, all chicks designated for grow out were removed from the climate respiration chambers, sexed, and transported to the grow out facilities, where they were housed in mixed sex groups of 6 males and 6 females. Both at D21 and D35 post hatch, 54 chickens per treatment were slaughtered, and their tibiatarsi and femurs were weighed and stored as described previously.

**Fig 2:**
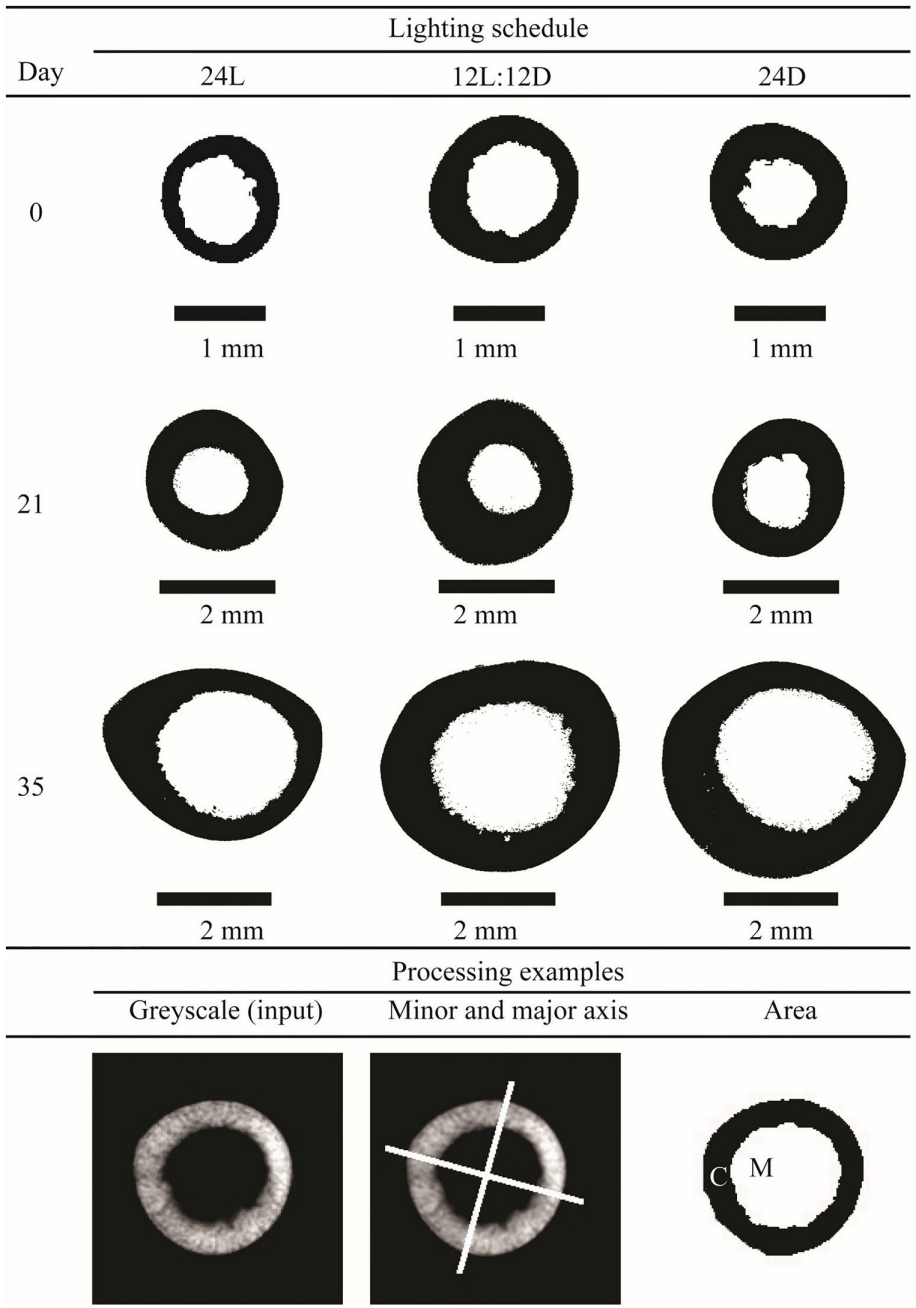
Examples of cross sections of tibiatarsus at the mid-diaphysis after reconstruction and thresholding of broilers incubated under 24L, 12L:12D, or 24D of white LED light from day 0 until hatching on D0, D21, and D35 post hatch. White bars in the minor and major axis examples shows how BoneJ determined the minor and major axis within a slide. C = cortical area, M = medullary area.

After thawing, the length, width (mediolateral diameter), and depth (craniocaudal diameter) of all tibiatarsi and femurs were measured using a digital calliper (Skandia, Ridderkerk, the Netherlands). The midpoint of 10 left sided tibiatarsi and femurs from D0, D21, and D35 (N = 90 tibiatarsi and femurs) were determined using the digital calliper, and the bone was sawed on both sides 0.9 cm away from the midpoint using a hacksaw, resulting in a 1.8 cm long bone segment from the middle of each bone. On D0, an additional 10 left sided tibiatarsi were used to determine the optimal procedure and data from these bones was added in the dataset, too. These segments were scanned using a Skyscan 1072 MicroCT scanner (Skyscan n.v., Aartselaar, Belgium). The X-ray source was set at 100 μA and 79 kV. Images were acquired with a 1.13° rotation step over an angular range of 180°, and with an exposure time of 3920 ms. See Fig 2 for examples of the femur’s and tibiatarsus’ cross sections on various days. A region of interest of 100 slides around the mid point was processed in NRecon (Bruker, Kontich, Belgium), and used for further analysis in ImageJ [26]. The plugin BoneJ [27] was used to determine cortical and medullary area, second moment of area around the minor and major axis (abbreviated here to minor and major area moment), and maximal and mean cortical thickness per slice. CT scan measurements were then averaged over slides per bone.

D35 legs (both left and right sided) were examined by a veterinarian for incidence of tibial dyschondroplasia, scored as an abnormality in the metaphysis of tibiotarsus or tarsalmetatarsis, characterized by an avascular cartilage plug at the bone’s proximal ends [28].

### Number of experimental animals

Embryos used between E8 and E14 were not considered to be experimental animals as per the guidelines of the Institutional Animal Care and Use Committee of Wageningen University, Wageningen, the Netherlands. The number of experimental animals was determined through a power analysis. The number of repetitions was calculated using the following formula:

$$N=(Z^{2}\times {standard\;deviation}^{2})\;÷Minimal\;difference\;for\;statistical\;significance$$

in which, for all calculations, a Z value of 1.96 was chosen.

Plasma melatonin concentration’s standard deviation was estimated to be 3 pg/ml [29] and the minimal difference for statistical significance was set at 7 pg/ml. This resulted in 5 chickens per treatment, per time point. At moment of hatch, various variables were measured but the largest variation was expected for tibia length and its standard deviation was estimated to be 4.42 mm at moment of hatching [30]. The minimal difference was set at 1.2 mm, resulting in 63 chicks per treatment All other measurements surrounding moment of hatch could also be performed in these chickens. For post hatch performance, body weight at D35 was expected to show the largest variation. The standard deviation for this was estimated to be 0.258 kg for a pen of 6 chicks. The minimal difference was set at 0.03 kg. This resulted in 9 pens of 6 chicks per treatment at slaughter.

### Statistical analysis

The statistical model used for analysis of the data of the current experiment primarily aimed to investigate the effect of incubation lighting schedules, taking time into account for pineal melatonin data. The overall model used was therefore:

$$Y_{i}=\mu +{Incubation}_{i}+\varepsilon i,$$ (1)

where Y_i_ = the dependent variable, *μ* is the overall mean, Incubation_i_ = Incubation lighting schedule (i = 24L, 12L:12D, or 24D), and *ε*_i_ = the residual error term. GH, IGF-I, embryonic bone data, and bone dimensions on D0 and D21 were analysed using generalized linear models in SAS (SAS Institute, Cary, NC, USA). Melatonin, bone dimensions on D35, and CT scan data were analysed using the Glimmix procedure (for fitting generalized linear mixed models), because not all data was normally distributed. The correct distribution type was determined by examining plots of the residuals. For melatonin data, an inverse Gaussian distribution was specified in the model. For tibia length and depth on D35, a lognormal distribution was specified in the model. Within the CT scan data, for E0 tibial cortical area and E0 tibial and femoral second moment of area (minor axis), D21 tibial and femoral cortical area and second moment of area (minor axis), and D35 femoral second moment of area (major axis) and mean cortical thickness, an inverse Gaussian distribution was specified in the model. For E0 tibial second moment of area (major axis), maximal cortical thickness, D21 femoral medullary area, D35 tibial second moment of area (minor and major axis), and tibial mean cortical thickness, a lognormal distribution was specified in the model. All other data were analysed assuming a normal distribution. Incidence of tibial dyschondroplasia was analysed using the Logistics procedure for logistic regression analysis of binary response data. For the analysis of embryonic melatonin data only, the model was extended with the moment of sampling (444, 450, 456, and 462 h of incubation) and its interaction with Incubation treatment.

Embryo or chicken was considered to be the experimental unit in the analysis of all data. For melatonin, one observation for 24L at 462h of incubation was considered an outlier (value 6x the standard deviation above the population’s average). This outlier was removed from analysis. Residuals were examined to verify model assumptions. Presented data are Least Square Means ±SEM. In all cases, differences were considered significant at *p ≤* 0.05.

## Results

### Plasma hormones

**Fig 3:**
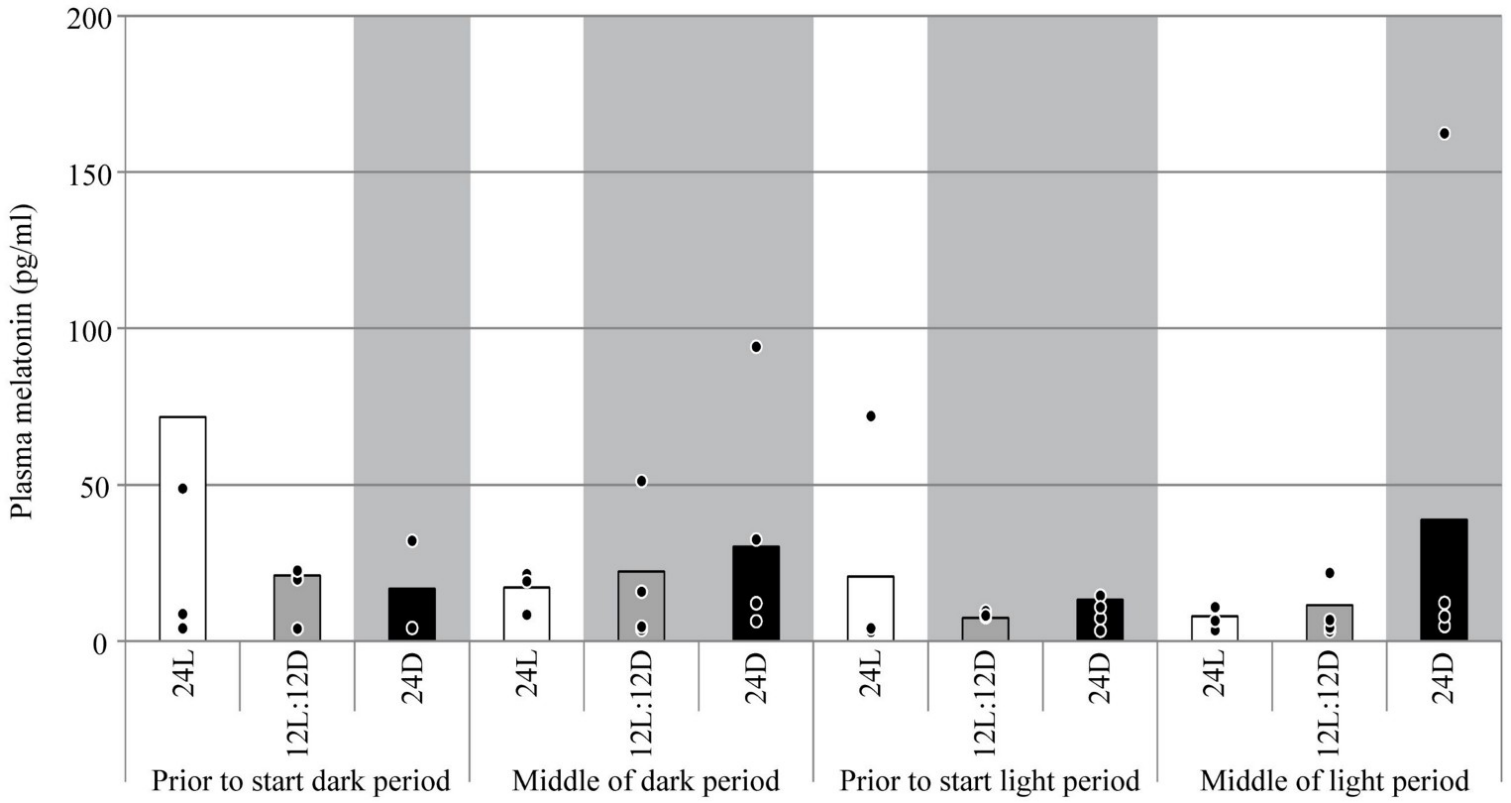
Plasma melatonin measured every 6 h between E18 +12h and E19 +6h in broiler embryos incubated under 24L, 12L:12D, or 24D of white LED light from set until hatch. Grey rectangle = dark period. • Individual observations. n = 5 per treatment, per sampling moment. x = Observation with actual value = 495 pg/ml, value not within axis range.

**Fig 4:**
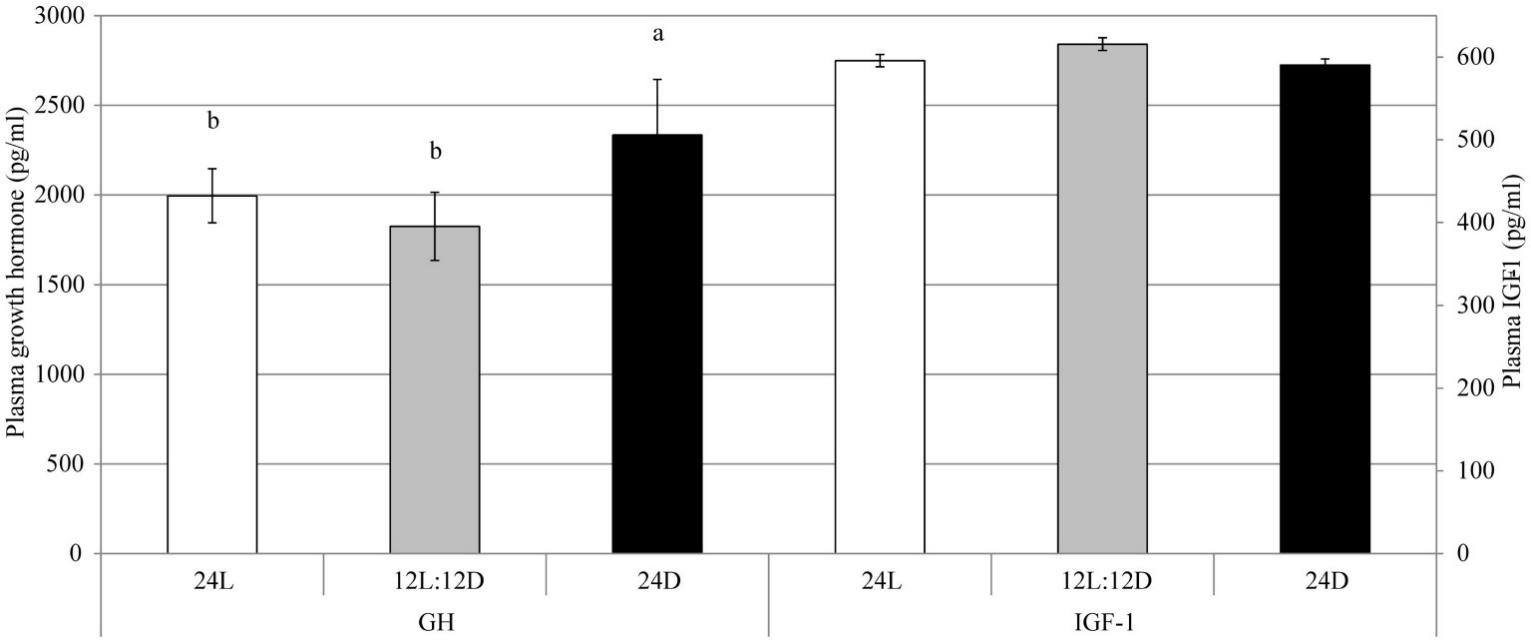
Plasma growth hormone (GH) and IGF-I ± standard errors at hatching in broiler chickens incubated under 24L, 12L:12D, or 24D of white LED light from day 0 until hatching. ^a,b^ Values within a hormone with different superscripts differ significantly at *p* ≤ 0.05. n = 50 chickens per treatment.

Plasma melatonin concentration between E18 +12h and E19 +6h was not affected by treatment (F = 0.53; *p*-value of the linear mixed model = 0.35), moment of sampling (F = 0.66; *p* = 0.59), or their interaction (F = 0.89; *p* = 0.85; Fig 3). GH at moment of hatching was higher for 24D than for 12L:12D (+21.8%) and 24L (+14.5%; F = 7.17; *p* = 0.002; Fig 4). IGF-I at moment of hatching was not affected by treatment (F = 0.90; *p* = 0.41).

### Embryonic bone development

Femur and tibiatarsus length were not affected by lighting treatment (F < 2.25; *p* > 0.13; Table 1). Femur width at E11 was higher for 24D than for 24L (+8.7%) and 12L:12D (+13.0%; F = 6.36; *p* = 0.006; Table 1). Tibiatarsus width at E8 was higher for 12L:12D than for 24L (+10.9%; F = 6.98; *p* = 0.004; Table 1). Tibiatarsus width at E11 was higher for 24D than for 24L (+2.3%) and 12L:12D (+6.1%; F = 11.10; *p* < 0.001). Femur and tibiatarsus width did not differ between treatments on other embryonic days (F < 3.38; *p* > 0.053).

**Table 1:** Femur and tibiatarsus length and width from E8 till E14 in broiler embryos incubated under 24L, 12L:12D, or 24D of white LED light from day 0 until hatching.

	n^1^	E8	E9	E10	E11	E12	E13	E14
	Femur length (mm)							
24L	10	4.33	5.45	7.16	8.21	9.90	11.33	12.83
12L:12D	10	4.40	5.27	6.92	8.39	10.02	11.26	12.57
24D	10	4.16	5.44	7.07	8.33	9.58	11.16	12.90
SEM		0.119	0.151	0.218	0.116	0.141	0.115	0.237
*p*-value		0.29	0.62	0.58	0.55	0.13	0.60	0.32
	Tibiatarsus length (mm)							
24L	10	4.73	6.53	8.84	10.34	12.82	14.86	17.07
12L:12D	10	4.71	6.58	8.49	10.50	12.92	14.83	17.01
24D	10	4.55	6.72	8.86	10.79	12.40	14.63	17.16
SEM		0.138	0.122	0.295	0.195	0.163	0.155	0.150
*p*-value		0.59	0.53	0.60	0.26	0.11	0.54	0.78
	Femur width (mm)							
24L	10	0.44	0.50	0.54	0.63^b^	0.83	1.01	1.23
12L:12D	10	0.44	0.50	0.53	0.60^b^	0.82	1.04	1.17
24D	10	0.44	0.50	0.54	0.69^a^	0.83	1.00	1.19
SEM		0.010	0.011	0.015	0.017	0.014	0.019	0.018
*p*-value		0.95	0.91	0.87	0.006	0.90	0.26	0.068
	Tibiatarsus width (mm)							
24L	10	0.41^b^	0.50	0.56	0.68^b^	0.82	1.00	1.32
12L:12D	10	0.46^a^	0.47	0.56	0.66^b^	0.81	1.06	1.29
24D	10	0.44^a^^b^	0.49	0.54	0.73^a^	0.84	1.01	1.24
SEM		0.014	0.008	0.019	0.011	0.019	0.020	0.031
*p*-value		0.004	0.064	0.75	<0.001	0.59	0.053	0.21

^a,b^Values within a day, within a measurement with different superscripts differ significantly at *p* ≤ 0.05.

^1^n = 10 chickens per treatment, per embryonic day.

**Fig 5:**
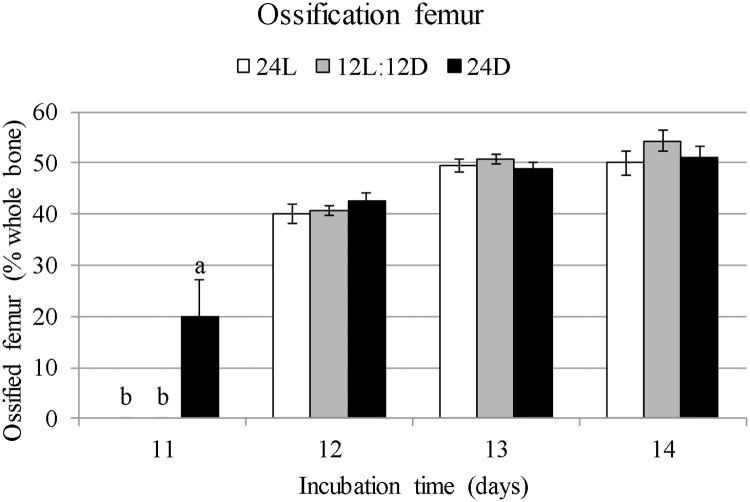
Ossified length of the femur as a percentage ±standard errors from E8 till E14 in broiler embryos incubated under 24L, 12L:12D, or 24D of white LED light from set until hatch. ^a,b^Values within a day with different superscripts differ significantly at *p* ≤ 0.05.

**Fig 6:**
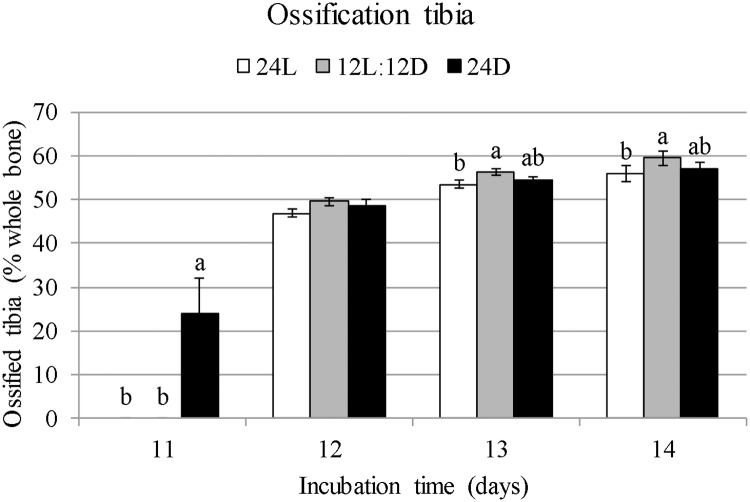
Ossified length of the tibiatarsus as a percentage ±standard errors from E8 till E14 in broiler embryos incubated under 24L, 12L:12D, or 24D of white LED light from set until hatch. ^a,b^Values within a day with different superscripts differ significantly at *p* ≤ 0.05.

The earliest ossification of the femur and tibiatarsus occurred on E11 (Figs 5 and 6). Of the 24D embryos, 6 out of 10 showed ossification while 0 out of 10 showed ossification of the 24L and 12L:12D embryos. On E12, all embryos had started ossification of the femur and tibiatarsus. Ossified percentage of the femur at E11 was higher for 24D than for 24L and 12L:12D (19.9% versus 0%; F = 15.77; *p* < 0.001; Fig 5). Ossified percentage of the femur did not differ between treatments on E12 (F = 1.32; *p* = 0.29), E13 (F = 1.85; *p* = 0.18), or E14 (F = 3.24; *p* = 0.057). Ossified percentage of the tibiatarsus at E11 was higher for 24D than for 24L and 12L:12D (+23.9% versus 0%; F = 15.77; *p* < 0.001). It did not differ between treatments on E12 (F = 2.13; *p* = 0.14). Ossified percentage was higher for 12L:12D than for 24L at E13 (+2.83%; F = 3.76; *p* = 0.040) and E14 (+3.46%; F = 3.53; *p* = 0.045), with 24D intermediate (Fig 6).

### Post hatch bone development

On day of hatching (D0), femurs were longer for 12L:12D than for 24L (+1.6%) and 24D (+1.5%; F = 4.73; *p* = 0.010; Table 2). Tibiatarsus weight was higher for 12L:12D (+6.5%) and 24D (+3.3%) than for 24L (F = 7.31; *p* = 0.001). Tibiatarsi were longer for 12L:12D (+2.0%) and 24D (+1.2%) than for 24L (F = 6.17; *p* = 0.003). Femur weight, depth, and width, and tibiatarsus depth and width did not differ between treatments (F < 2.60; *p* > 0.08).

**Table 2:** Femur and tibiatarsus weight, length, width, and depth on D0, D21, and D35 post hatching in broilers incubated under 24L, 12L:12D, or 24D of white LED light from day 0 until hatching.

		Femur				Tibiatarsus			
	n^1^	Weight (g)	Length (mm)	Depth (mm)	Width (mm)	Weight (g)	Length (mm)	Depth (mm)	Width (mm)
		Day 0 post hatch							
24L	60	0.197	21.91^b^	1.52	1.54	0.29^b^	30.07^b^	1.41	1.51
12L:12D	60	0.204	22.26^a^	1.53	1.55	0.31^a^	30.67^a^	1.43	1.54
24D	60	0.201	21.93^b^	1.54	1.55	0.30^a^	30.45^a^	1.44	1.53
SEM		0.002	0.09	0.009	0.01	0.004	0.12	0.01	0.01
*p*-value		0.11	0.010	0.25	0.92	0.001	0.003	0.08	0.22
		Day 21 post hatch							
24L	54	4.44	50.29^b^	6.27	5.85	6.35	72.00^b^	5.12	5.59
12L:12D	54	4.47	51.43^a^	6.27	5.79	6.47	73.57^a^	5.16	5.55
24D	54	4.52	51.20^a^	6.41	5.94	6.53	73.18^a^	5.14	5.63
SEM		0.08	0.28	0.06	0.05	0.12	0.40	0.06	0.05
*p*-value		0.79	0.010	0.18	0.12	0.53	0.020	0.92	0.52
		Day 35 post hatch							
24L	54	10.64^b^	68.62	9.18^b^	8.76	14.34	99.71^b^	7.07	8.44
12: 12D	54	11.28^a^	69.02	9.25^a^^b^	8.85	14.88	101.27^a^	7.12	8.51
24D	54	10.78^a^^b^	68.78	9.49^a^	8.85	14.71	100.69^a^^b^	6.96	8.46
SEM		0.175	0.299	0.066	0.063	0.225	0.404	0.072	0.077
*p*-value		0.023	0.61	0.003	0.52	0.23	0.023	0.26	0.81

^a,b^Values within a day, within a measurement with different superscripts differ significantly at *p* ≤ 0.05.

^1^n = 50 chickens per treatment at hatching and 54 per treatment on day 21 and day 35 post hatch

On D21 post hatch, femurs were longer for 12L:12D (+2.2%) and 24D (+1.8%) than for 24L (F = 4.77; *p* = 0.010). Tibiatarsi were longer for 12L:12D (+2.1%) and 24D (+1.6%) than for 24L (F = 4.05; *p* = 0.020). Tibiatarsus and femur weight, depth, and width did not differ between treatments (F < 2.13; *p* > 0.12).

On D35 post hatch, femur weight was higher for 12L:12D than for 24L (+5.7%; F = 3.85; *p* = 0.023), with 24D intermediate. Femur depth was higher for 24D than for 24L (3.3%) and 12L:12D (+2.5%; F = 6.00; *p* = 0.003). Tibiatarsi were longer for 12L:12D than for 24L (+1.5%), with 24D intermediate (F = 3.82; *p* = 0.023). Femur length and width, and tibiatarsus weight, depth, and width did not differ between treatments (F < 1.48; *p* > 0.23).

Incidence of tibial dyschondroplasia was higher for 24L (8.3% of all tibiatarsi) than for 12L:12D (1.0% of all tibiatarsi), with 24D intermediate (1.9% of all tibiatarsi; *p* = 0.046).

### CT scans

On D0 post hatch, cortical area of the femur was not affected by treatment (F = 1.9; *p* = 0.17; Table 3). Cortical area of the tibiatarsus was higher for 12L:12D (+10.0%) and 24D (+13.6%) than for 24L (F = 4.3; *p* = 0.019). Medullary area of the femur and tibiatarsus were not affected by treatment (F < 2.87; *p* > 0.073). The minor area moment of the femur was higher for 24D than for 12L:12D (+21.6%; F = 3.48; *p* = 0.044). The minor area moment of the tibiatarsus was higher for 24D and 12L:12D than for 24L (both +19.4%; F = 4.34; *p* = 0.018). The major area moment of the femur and tibiatarsus, maximal cortical thickness of the femur and tibiatarsus, and mean cortical thickness of the femur did not differ between treatments (F < 2.15; *p* > 0.053). Mean cortical thickness of the tibiatarsus was higher for 12L:12D (+17.2%) and 24D (+14.3%) than for 24L (F = 4.92; *p* = 0.011).

**Table 3:** Femur and tibiatarsus cortical and medullary area, minor and major area moment, and maximal and mean cortical thickness at D0, D21 and D35 post hatching in broilers incubated under 24L, 12L:12D, or 24D of white LED light from set until hatch.

		Cortical area (mm^2^)		Medullary area (mm^2^)		Second moment of area Minor axis (mm^4^)		Second moment of area Major axis (mm^4^)		Maximal cortical thickness (mm)		Mean cortical thickness (mm)	
	n^1^	Femur	Tibia-tarsus	Femur	Tibia-tarsus	Femur	Tibia-tarsus	Femur	Tibia-tarsus	Femur	Tibia-tarsus	Femur	Tibia-tarsus
		Day 0 post hatch											
24L	10	1.28	1.08^b^	2.31	2.14	0.31^a^^b^	0.25^b^	0.34	0.29	0.44	0.32	0.30	0.24^b^
12L:12D	10	1.21	1.20^a^	2.26	2.29	0.29^b^	0.31^a^	0.32	0.34	0.40	0.36	0.29	0.29^a^
24D	10	1.36	1.25^a^	2.47	2.28	0.37^a^	0.31^a^	0.39	0.35	0.44	0.35	0.31	0.28^a^
SEM		0.055	0.046	0.064	0.060	0.020	0.016	0.021	0.018	0.021	0.0127	0.016	0.011
*p*-value		0.17	0.019	0.073	0.14	0.044	0.018	0.053	0.058	0.29	0.13	0.55	0.011
		Day 21 post hatch											
24L	10	20.40	17.91	10.06	6.26	53.58	38.76	70.30	48.95	1.45	1.46^b^	0.81	1.05
12L:12D	10	19.36	18.05	9.91	6.92	49.35	40.40	65.78	51.48	1.43	1.60^a^	0.82	1.10
24D	10	18.47	16.07	9.88	6.69	46.09	33.43	60.00	43.18	1.51	1.41^b^	0.90	1.02
SEM		0.747	0.864	0.703	0.575	4.380	3.570	5.896	4.874	0.062	0.047	0.056	0.052
*p*-value		0.21	0.22	0.98	0.71	0.49	0.38	0.48	0.49	0.64	0.033	0.47	0.58
		Day 35 post hatch											
24L	10	28.60	22.93^b^	28.37	18.07^b^	162.35	86.82^b^	219.10	129.83	1.35	1.77^a^	0.82	1.12^a^
12L:12D	10	26.29	27.84^a^	29.45	19.77^a^^b^	151.62	121.29^a^^b^	197.83	180.99	1.32	1.70^a^	0.82	1.06^a^^b^
24D	10	28.95	27.74^a^	30.24	23.42^a^	170.50	141.76^a^	231.92	200.53	1.30	1.44^b^	0.75	0.91^b^
SEM		1.110	1.376	1.225	1.440	11.139	14.703	16.097	20.044	0.056	0.065	0.055	0.061
*p*-value		0.203	0.030	0.564	0.042	0.495	0.046	0.333	0.056	0.852	0.003	0.586	0.048

^a,b^Values within a day, within a measurement with different superscripts differ significantly at *p* ≤ 0.05.

^1^n = 10 bones (tibiatarsus or femur) per treatment, per day, with the exception of D0 tibiatarsi (n = 20 bones).

See S1 Dataset for the dataset.

On D21 post hatch, cortical and medullary area, minor and major area moments, and mean cortical thickness of the tibiatarsus and femur, as well as maximal cortical thickness of the femur were not affected by treatment (F < 1.66; *p* > 0.21). Maximal cortical thickness of the tibiatarsus was higher for 12L:12D than for 24L (+8.8%) and 24D (+11.9%; F = 3.93; *p* = 0.033).

On D35 post hatch, cortical area of the tibiatarsus was 17.3 to 17.7% higher for 12L:12D and 24D than for 24L (F = 4.02; *p* = 0.030). Medullary area of the tibiatarsus was 22.8% higher for 24D than for 24L, with 12L:12D intermediate (F = 3.6; *p* = 0.042). Minor area moment of the tibiatarsus was 38.8% higher for 24D than for 24L, with 12L:12D intermediate (F = 3.48; *p* = 0.046). Maximal cortical thickness of the tibiatarsus was 15.4 to 18.9% higher for 24L and 12L:12D than for 24D (F = 7.26; *p* = 0.003). Mean cortical thickness of the tibiatarsus was 19.5% higher for 24L than for 24D, with 12L:12D intermediate (F = 3.43; *p* = 0.048). Cortical and medullary area, minor and major area moment, and maximal and mean cortical thickness of the femur and major area moment of the tibiatarsus did not differ between treatments (F < 3.22; *p* > 0.056).

An overview of all bone variables significantly affected by treatment can be seen in Table 4.

**Table 4:** Overview of femur and tibiatarsus measurements (embryonically, at hatching, or at D21 or D35 post hatch) found to be significantly decreased (*p* < 0.05) by treatment compared to another treatment for broilers incubated under 24L, 12L:12D, or 24D of white LED light throughout incubation.

Phase	Bone	Measurement	Decreased for	Compared to
Embryonic	Femur	Width E12	24D	12L:12D and 24L
	Tibiatarsus	Width E8	24L	12L:12D
		Width E11	24L and 12L:12D	24D
		Ossification E13	24L	12L:12D
		Ossification E14	24L	12L:12D
Hatching	Femur	Length	12L:12D	12L:12D
		Minor area moment^1^	24L and 24D	24D
	Tibiatarsus	Weight	24L	12L:12D and 24D
		Length	24L	12L:12D and 24D
		Cortical area	24L	12L:12D and 24D
		Minor area moment^1^	24L	12L:12D and 24D
		Mean cortical thickness	24L	12L:12D and 24D
D21	Femur	Length	24L	12L:12D and 24D
	Tibiatarsus	Length	24L and 24D	12L:12D
		Maximal cortical thickness	24L and 24D	12L:12D
D35	Femur	Weight	24L	12L:12D
		Depth	24L	24D
	Tibiatarsus	Length	24L	12L:12D
		Cortical area	24L	12L:12D and 24D
		Medullary area	24L	24D
		Minor area moment^1^	24L	24D
		Maximal cortical thickness	24L and 12L:12D	24D
		Mean cortical thickness	24L	24D
		No tibial dyschondroplasia	24L	12L:12D

^1^Minor or major area moment—second moments of area around the minor and major axis.

## Discussion

Ossification of embryonic chicken bones starts at the mid diaphysis of the bone in the primary ossification centre [7]. In the current experiment, ossification of the tibiatarsus and femur started approximately a day sooner for embryos exposed to 24D than to 12L:12D and 24L. For 6 out of 10 embryos from the 24D group, ossification of the tibiatarsus and femur started on E11. For all other 24D embryos, and all embryos from 12L:12D and 24L, it started on E12. Although the onset of ossification was earlier for 24D, the 12L:12D and 24L treatments no longer differed on E12. On E13 and E14, ossification of the tibiatarsus of 12L:12D was higher than that of 24L, suggesting that rate of ossification in the tibiatarsus had increased for 12L:12D compared to 24L from E13 onward. These results suggest that 24D accelerated onset of ossification, and an incubation lighting schedule of 12L:12D increased osteoblast activity by E13 in the tibiatarsus of broiler embryos more than continuous light.

By the time the chickens hatched, 12L:12D and 24D had longer and heavier tibiatarsi than 24L and 12L:12D had longer femurs than both 24L and 24D. Increase of bone length is mostly dependent on the rate with which hypertrophic cartilage cells are produced at the bone’s epiphysis [31]. The cartilage is then replaced by bony tissue through endochondral ossification [10]. It appears that 24L may decrease both ossification at the primary ossification centre, and bone length growth at the epiphyseal plates, compared to 12L:12D, with 24D intermediate.

Osteoblast formation is said to be stimulated by melatonin, which is released in dark periods [13]. It could therefore be hypothesized that incubation under 24L results in lower basal melatonin levels than 24D or 12L:12D, which provide darkness. However, in the current experiment, plasma melatonin concentrations between E18 +12h and E19 +6h did not differ between treatments or phase within the light-dark schedule. This is contrary to results found by [2,32]. [32] incubated eggs under various lighting schedules, such as 16L:8D and 8L:16D, and found a clear dark-light dependent rhythm of plasma melatonin from E18 onward, but it was less pronounced than pineal melatonin concentrations. It is possible that a rhythm in melatonin release would have been observed if melatonin concentrations in the pineal gland had been measured in the current experiment. Growth hormone and IGF-I are known to stimulate chondrocyte proliferation in epiphyseal plates [18,19]. Growth hormone level at hatching was found to be higher for 24D than for 12L:12D and 24L, and IGF-I level at moment of hatching was not affected by treatment. However, growth hormone was analysed for chicks from the peak of the hatch window, and this coincided with 12L:12D’s light period. This may explain why growth hormone levels were comparable for 24L and 12L:12D. Unfortunately, no data is available on growth hormone or IGF-1 for 12L:12D chicks in their dark period, but it is possible that growth hormone levels would have been higher in darkness. The results of this experiment do not clarify the endocrine mechanism behind the stimulatory effect of 12L:12D and, to a lesser degree, 24D on embryonic bone development compared to 24L.

Microstructure of the tibiatarsus differed between treatments at hatching of the chickens. 12L:12D and 24D both had a larger tibiatarsus cortical area, minor area moment, and mean cortical thickness than 24L. The negative effect was still present at slaughter. At slaughter on D35, higher tibiatarsal cortical area (but not thickness) was found for 12L:12D and 24D than for 24L. Tibiatarsus medullary area and minor area moment were higher for 24D than for 24L. Additionally, femur weight (but not length) was higher for 12L:12D than for 24L, and tibiatarsus length (but not weight) was still higher for 12L:12D than for 24L at D35. According to [12], bone cortical area, second moment of area, and bone size itself can predict up to 70 to 80% of whole bone strength. A higher second moment of area indicates greater resistance to bending. In adult humans, the risk of stress fractures in both the tibiatarsus and femur has been found to be increased with decreasing moments of inertia [33]. The decrease in the values of the bone parameters in the 24L incubated chickens therefore suggests that these bones were not only smaller but also weaker at slaughter than those of 12L:12D and 24D.

The tibiatarsi of 12L:12D showed a lower incidence of tibial dyschondroplasia compared to 24L. Tibial dyschondroplasia’s aetiology lies in poor ossification of the epiphyseal plates, characterized by an avascular lesion in the tibiatarsal head [34]. It is a form of osteochondrosis, which is an abnormality in endochondral ossification [28]. It is not known how incubation under 12L:12D reduced incidence of tibial dyschondroplasia, but it can be speculated that applying a light-dark schedule stimulated vascularization [35] or ossification [34] at the epiphyseal plate.

The current experiment suggests that continuous light throughout incubation had a detrimental effect on embryonic bone development. This effect was still present at slaughter, with larger, stronger bones for 24D and 12L:12D, and less tibial dyschondroplasia at D35 post hatch for 12L:12D than for 24L. These results suggest that darkness is essential for chicken embryonic bone development, but applying a lighting schedule may stimulate leg bone development and health further. The tibiatarsus seems especially susceptible to stimulation by incubation lighting schedules. The involvement of hormones in the stimulatory effect of a light-dark rhythm during incubation, however, cannot be explained based on the current experiment. In chickens, we can conclude that continuous light during incubation negatively influences embryonic bone development with possible long term effects on leg bone health compared to continuous dark or applying a lighting schedule.

## Supporting information

S1 Dataset Data for embryonic ossification (“Cartilage” tab), embryonic plasma melatonin (“Melatonin” tab), growth hormone and IFG-1 at hatching (“GH&IGF-1” tab), post hatch bone dimensions (“BoneData” tab), and CT scan data (“CT-scans” tab). (XLSX)
